# Digital Job Demands and Resources: Digitization in the Context of the Job Demands-Resources Model

**DOI:** 10.3390/ijerph20166581

**Published:** 2023-08-15

**Authors:** Alexander Scholze, Achim Hecker

**Affiliations:** 1 Institute for Management and Economics in Health Care, UMIT TIROL—Private University for Health Sciences and Health Technology, 6060 Hall in Tirol, Austria; achim.hecker@dbuas.de; 2 Department of Business Administration, DBU Digital Business University of Applied Sciences, 10999 Berlin, Germany

**Keywords:** digitization, digital information and communication technology, job demands-resources model, work design, working conditions

## Abstract

This study comprehensively investigates the effects of digitization in the workplace, with a specific focus on white-collar employees, using the job demands-resources (JD-R) model as a theoretical framework. By examining the intricate interplay between digital job demands and digital job resources, the research offers valuable insights to help organizations navigate the complexities caused by technological advancements. Utilizing a qualitative triangulation approach, the research combines a systematic literature review with a thematic analysis of 15 interdisciplinary expert interviews. Thereby, the study establishes a robust theoretical foundation for exploring stress, motivation, and the organizational consequences arising from integrating technology in the workplace. The JD-R model is extended to incorporate digital job demands and resources, enabling a thorough examination of both the positive and negative aspects of digitization within organizations. Moreover, the study highlights the necessity for the consistent adaptation of the JD-R model across diverse job contexts in the ever-evolving digital landscape. It advocates for organizations to effectively leverage digital resources and proactively manage job demands, aiming to transform digitization into a valuable job asset while preventing the onset of overwhelming burdens. In conclusion, the research encourages organizations to embrace the vast potential of digitization while prioritizing digital health in the workplace.

## 1. Introduction

Digitization describes the technical process of converting analog signals into a digital form [1]; in the work context, this promotes the use of digital information and communication technologies (DICT) [2]. Digitalization, as a consequence, describes the socio-technical phenomena and processes of adopting and using these DICT [1] in organizations, while digital transformation describes a cultural shift that is necessary to integrate the required changes [3]. In this context, the increasing use of DICT has significantly impacted working conditions in various workplaces and poses challenges for organizations. An intensification of digitization, driven by COVID-19, has brought into sharp focus both the beneficial nature of DICT and their potential dark side [4]. Technostress, the stress experienced by users of DICT [5], is already a well-known phenomenon in this context, but it takes on a new dimension considering the issue of how we design sustainable and health-oriented workplaces in the future [6,7,8,9,10]. To find the right concepts for organizations to deal with digitalization, there is a growing need to understand the impact of digitalization on work-related stress and the motivation to design workplaces to meet the changing working conditions within the digital transformation process [11,12,13,14,15]. The impact of digitization in the work context is ambiguous, as it often affects work demands and work resources simultaneously; therefore, a comprehensive understanding of the impact of digitization on working conditions is still lacking. This knowledge gap partly stems from the absence of an integrated theoretical model that considers the ambiguous effects of digitization in the workplace. Therefore, there is an urgent requirement for a robust theoretical and conceptual basis to guide future empirical studies, comprehend the changes in working conditions brought about by digitization, gain a deeper understanding of its impact, and develop work concepts that protect employees’ mental health in the digital age.

### 1.1. Theoretical Background

Existing theoretical models in organizational psychology—such as the two-factor model [16], job characteristics model [17], demand-control model [18], or effort-reward imbalance model [19]—have often been criticized for their one-sidedness, simplification, lack of context, and static character, which ignores the dynamic nature of work [20,21].

The job demands-resources (JD-R) model integrates elements from these established models; by combining different perspectives, it provides a more holistic view of the work environment [22].

**Figure 1:**
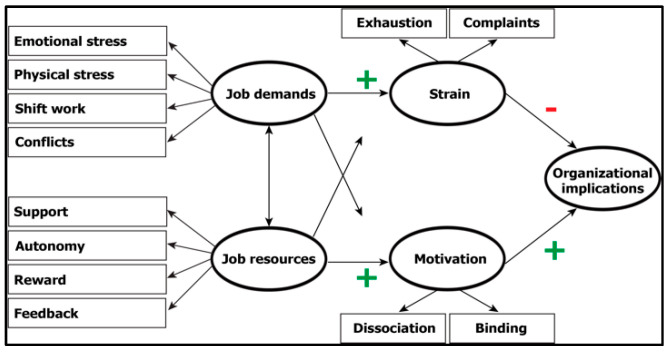
The JD-R model presented by Demerouti and Nachreiner [33].

The JD-R model, developed by Demerouti et al. [23], was intended to examine burnout and the life satisfaction status of employees in the workplace [24]. Although the fields of application of the JD-R model have expanded, it still provides new insights into the study of burnout [25]. One reason for this constant actuality is that the JD-R model considers working conditions to be dynamic, which can change owing to various factors. This adaptive quality helps accommodate changing work realities, for example, those caused by COVID-19 [24]. It recognizes that job demands and resources may evolve, impacting employees’ experiences and outcomes [22]. For example, a study of airline cabin crew members showed the influence of intercultural communication skills and service attitude on the relationship between cultural intelligence and lower cabin crew anxiety [26]. The JD-R model accounts for complexity by integrating a wide variety of factors; it is dynamic and flexible to adapt to different work contexts, and it focuses bilaterally on the positive and negative outcomes of workplaces [22]. Kwon and Kim also address the dynamic of job demands and job resources by examining the relationship between employee engagement and innovative behavior in the context of the JD-R model [27]. A substantial body of research supports the generalizability of the model across populations, workplaces, cultures, and countries [28,29,30,31,32]. These studies corroborate the validity and generalizability of the model and its constructs, particularly regarding their effects on strain and motivation in organizations. Figure 1 shows the JD-R model in a version presented by Demerouti and Nachreiner [33]. The model has undergone continuous refinement since its initial publication and has been widely used in increasingly different work scenarios [20,22,23,33,34,35]. It provides actionable insights for organizations to improve job design; by 2007, the model had been applied in over 130 organizations, aiding human resources practitioners and consultants in diagnosing demoralization issues within their respective workplaces [22].

The JD-R model combines two dual processes: the health-impairment process and the motivation-driven process [21]. Thereby, it emphasizes the interaction effects between job demands and resources. It recognizes that a high number of job resources can buffer the negative effects of high job demands, thereby mitigating potential stress and burnout [33]. Job demands represent a job’s physical, psychological, social, and organizational aspects, which generally require prolonged physical or psychological exertion [23]. Job resources define working conditions that meet three criteria: being functionally necessary for achieving job-related goals, reducing the physical and psychological costs of job demands, and stimulating personal growth and development [23]. In the JD-R model, job demands are associated with decreased health, whereas job resources are primarily associated with increased motivation [33].

Overall, the described features of the JD-R model provide an optimal theoretical framework to investigate aspects of digitization related to psychological stress and motivation in the work context.

Although digitization was not originally the focus of the JD-R model, some studies have incorporated it within the JD-R framework. This study focuses on digitization as a job demand on the one hand—technology-based demands [36] or IT-related job demands [37]—and on the other hand as job resources such as computer self-efficacy [38] and IT mindfulness [39].

Another group of publications have examined the relationship between digitization and mental stress based on the JD-R model. These studies have investigated aspects such as “Off-Job Technology [40]”, “Intensive Internet Use [41]”, “Working Remotely [42]”, and “Flexible Job Designs [43]”.

All these publications already show different approaches to using the JD-R model as a theoretical framework to examine the impact of changes through digitization in the work context. An extension of the JD-R model that captures the ambiguous effects of digitization on both job demands and resources is the next step in providing a holistic framework for understanding the impact of digitization in different work scenarios. Currently, such a structured and comprehensive view of the benefits and drawbacks of digitization in the workplace is lacking.

### 1.2. Research Gap and Aim of the Study

We aim to bridge this gap by extending the JD-R model to gain a more comprehensive understanding of the ambiguous effects of digitization on job demands and resources. Based on the definition of these variables, we aim to build a theoretical framework for further quantitative studies in different work scenarios. To achieve this aim, this study extends the well-established JD-R model to develop a theoretical framework that incorporates the ambiguous aspects of digitization based on the work scenario of white-collar employees. The guiding research question is “How can digitization be comprehensively and systematically integrated into the JD-R model?” By integrating aspects of digitization within the work scenario of white-collar employees, we take the theoretical extension of the JD-R model based on a concrete practical example. This practice-based extension of the model serves as a valuable theoretical foundation for preventing mental stress in digitized working conditions and guides future research in this area. Moreover, it is intended to support organizations in shaping the working environment of employees in the context of digitalization in a way that prevents health problems.

## 2. Materials and Methods

The qualitative triangulation approach used in this study combines the findings from a systematic literature review with guided expert interviews. This approach enables us to examine the main effects of digitization on white-collar employees in the workplace context and classify them according to the model’s variables of job demands and resources. The definitions of these model variables provide an appropriate foundation for methodologically organizing and exploring the effects.

**Figure 2:**
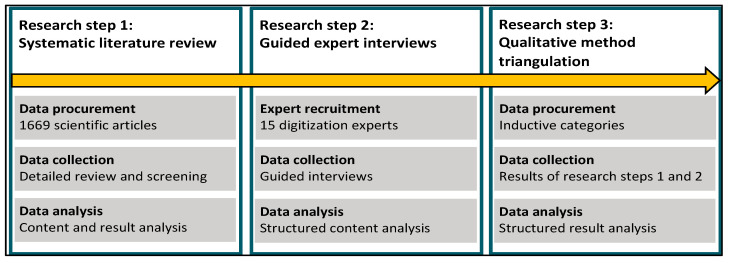
Schematic of the research design.

The research process is detailed in Figure 2. First, a systematic literature review was conducted to identify the effects of digitization in the workplace, which were then classified as job demands or job resources based on the JD-R model definitions. Second, we conducted guided interdisciplinary expert interviews to gain deeper insights into the experiences of white-collar employees. The results of these interviews were also analyzed for the effects of digitization and categorized based on the criteria of the JD-R model. Finally, in the third research step, we performed a triangulation of both methods to compare the identified effects from the previous research steps and determine the most relevant effects for discussion. This approach facilitates a comprehensive understanding of the impact of digitization on job demands and resources, providing examples of job demands and job resources stemming from digitization.

### 2.1. Systematic Literature Search (Research Step 1)

We conducted a systematic literature search between November 2020 and March 2021 using interdisciplinary databases (EBSCO Complete, PsyInfo, Scopus) to capture a comprehensive state of research in the field. The search utilized the following keywords: “(psy* AND strain OR stress OR resource) AND (digi* OR virtu* OR ICT* or information and communication technology) AND (Job* OR job OR employment OR occupation OR demand*)”. The following inclusion criteria were applied: publication period, 2000–2021; source type, academic journal; and language, German or English. A total of 1669 scientific articles were identified.

These articles were screened to identify those relevant to the research context. We exclusively focused on publications that examined the effects of digitization in the workplace. Publications with a different research focus were excluded from the structured screening process. The following categories of publications were excluded:

- Other organizational topics, such as leadership, agility, and mobbing (n = 263);
- Digitization in the context of personal life (n = 248);
- Digitization projects and process optimization (n = 174);
- Human–machine interaction (n = 158);
- Health applications, social media, and platforms (n = 131);
- Mental strain or stress without digitization aspects (n = 107);
- Other topics (n = 61).

**Figure 3:**
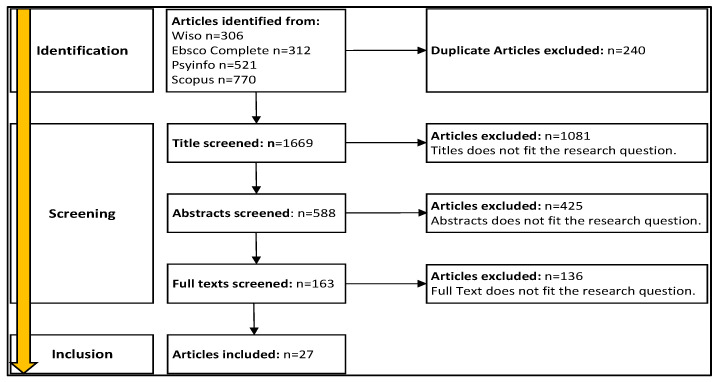
Screening process of the systematic literature review.

Figure 3 illustrates the steps involved in the screening process. A full-text analysis was conducted on 163 articles, with a focus on examining the methods, results, and conclusion sections in relation to the research question.

A total of 27 publications were analyzed to identify the effects of digitization, which can be attributed to job demands and resources. The identified effects of digitization were classified based on the definitions of the model variables—job demands and job resources—to determine their overall frequency of occurrence.

### 2.2. Guideline-Based Expert Interviews (Research Step 2)

We conducted guided interviews with 15 experts between June and September 2021. We formed an interdisciplinary expert panel comprising three expert groups: science professors researching digitization in the workplace, senior executives with management experience in digitizing the workplace, and senior medical officers focusing on the use of DICT in the workplace. Theoretical sampling [44] was applied, and experts were selected based on their expected contributions to generating new information and insights for development.

The interdisciplinary composition of the expert panel provided a range of perspectives on the research subject. This allowed the inclusion of many aspects and improved the transferability of the results [45].

Based on the research question and the current state of research on the JD-R model, we developed a theory-based interview guide focusing on the model variables, job demands and job resources, using definitions from the JD-R model.

To ensure the quality of the study, we conducted a pretest and made minor adjustments based on the feedback received before conducting online interviews with experts through a web conference. Before the interviews, the participants received written information about the study, including its purpose, format, duration, confidentiality, and data usage. The expert-guided interviews were transcribed verbatim, and the resulting transcripts were analyzed using structured theory-based content analysis [46]. The analysis involved extracting structures from the material guided by the JD-R model, especially based on the definitions of job demands and job resources. The job demands and resources variables of the JD-R model were used for the systematic theory-based processing of expert interviews, which formed a deductive component of the coding strategy [47]. The data were analyzed and processed using MAXQDA (Release 20.4.1; VERBI GmbH) [48]. To ensure the quality of the evaluation results, a second researcher verified the coding, which yielded an interrater reliability of 91%. The transcripts were not returned to the experts after the completion of the interviews to maintain confidentiality.

### 2.3. Qualitative Triangulation (Research Step 3)

Finally, we triangulated our results to increase the confidence and validity of the research findings [48,49]. At this point, we examined how often the different effects of digitization, classified as digital job demand or digital job resource, were described in the literature and guided interviews, thereby structuring and consolidating the results. By assessing the frequency of these effects, we gained insight into the significance of each factor within our research field, which allowed us to focus on the most relevant effects in the context of white-collar employees.

**Figure 4:**
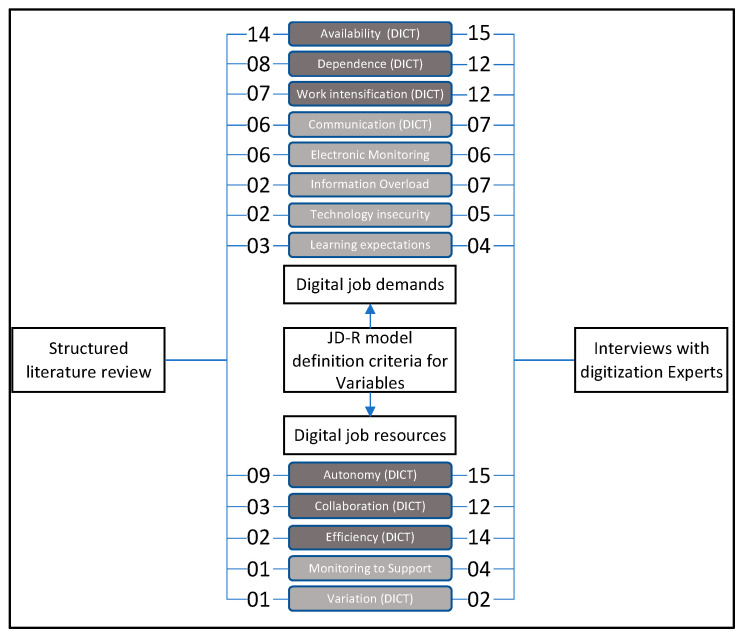
Categories are presented in descending order based on a calculated score, derived from both the literature review and expert interviews. The numbers represent the frequency of occurrence for each category in the literature review and expert interviews.

Figure 4 shows the triangulated results. We calculated a combined score by adding the results from steps 1 and 2. In our further research, to consider theoretical implication and practical impact, we focused on the three digital job demands and digital job resources with the highest relevance. This scenario of digital working conditions provided the basis for extending the JD-R model.

The significance of each digital job demand or resource can vary depending on the work scenario. For our work scenario with white-collar employees, we primarily focused on three digital job demands: availability (score: 29), dependency (score: 20), and work intensification (score: 19). Furthermore, we emphasized three constructs as digital job resources: autonomy (score: 24), collaboration (score: 15), and efficiency (score: 16). These constructs were considered crucial for understanding the impact of digitization in the workplace. These effects of digitization in the work context have a high relevance in the expert interviews and literature research.

## 3. Results

The established definitions of the JD-R model’s variables, job demands and job resources, facilitate a structured approach to understanding the effects of digitization within a work context. It enables the consideration of reciprocal effects within the model framework.

This structured extension of the JD-R model offers a theoretical foundation for investigating the impact of digitization on strain and motivation. Furthermore, it enables the classification of other effects of digitization and future technologies as either job demands or job resources, facilitating their examination in terms of their influence on the work context and their impact on psychological strain.

### 3.1. Digital Job Demands

The identified constructs represent key aspects that affect the working conditions of white-collar employees using DICT. Essentially, digital job demands represent the consequences of using DICT, which influence the work context and require prolonged psychological exertion.

#### 3.1.1. Availability via DICT

The use of DICT has changed employee cooperation and communication. DICT enables employees to be constantly accessible. Previous studies have discussed this aspect and its effects, particularly the blurring of boundaries between personal and work lives.

Day et al. [2] identified the availability via DICT as a job demand. Employees must create individual strategies to cope with this effect of digitization [50]. The consequences of availability are permeability, flexibility, and spillover of work into employees’ personal lives. These spillovers can subsequently lead to burnout, job dissatisfaction, and even resignation [51]. Rohwer et al. [52] showed that a higher degree of virtuality coincides with higher levels of boundarylessness. In this context, employees’ boundary management becomes a vital aspect of their individual strategies to manage the intrusion of work into their private lives [53,54]. Conducting work during off-work hours [40] has been shown to result in work–life conflicts [54,55].

Continuous availability has been found to increase the pressure on employees regarding their expectations of promptly responding to emails. These expectations can have a long-term impact on employees’ mental health [56].

The expert interviews supplement the findings from the literature review and classify availability via DICT as having a major influence on job demands. All 15 experts stated that availability via DICT increases job demand.

- “Being constantly available everywhere creates an entirely new pressure in the workplace.” (Expert 7)
- “Digitization is highly stressful for employees because they can be reached instantly.” (Expert 9)
- “The constant availability through digital media is like being omnipresent.” (Expert 1)
- “You have this constant availability, 24/7.” (Expert 14)
- “If taken to the extreme, it results in being available 24/7.” (Expert 15)

The literature and experts highlighted the diversity of the effects of availability in the workplace. Owing to the possibly wide range of consequences of availability via DICT, this aspect of digitization is highly relevant to the study of the working environment. Therefore, availability is considered a key factor in extending the JD-R model to integrate the effects of digitization.

#### 3.1.2. Dependence on DICT

The growing reliance on DICT in the workplace has led to work interruptions caused by DICT issues, which can negatively affect work performance and increase psychological stress [43,56,57]. Furthermore, DICT problems, such as technology malfunctions [2], can further increase employee frustration [58]. Overall, extensive utilization of DICT can result in individuals encountering more DICT-related challenges. This effect is described in terms of the dependence of digital job demand on DICT.

Salmela-Aro and Upadyaya [59] highlighted the importance of employees with high resilience because resilience is a buffer between task dependence and job burnout. They recommended the implementation of an integrative lifespan approach to address the issue of DICT dependence.

Twelve experts identified the increasing reliance on DICT in the workplace as a crucial digital job demand due to its numerous consequences that impact working conditions. For instance, technical issues can result in work interruptions, significantly affecting job requirements and leading to an increased workload, especially in cases of high DICT dependency.

- “If the computer breaks now, the task cannot be completed.” (Expert 12)
- “In such scenarios, employees get stressed when something doesn’t function properly technically…” (Expert 5)
- “Being disrupted from the work routine reveals our reliance on digitization.” (Expert 3)
- “Technology sometimes demands excessive attention, which disrupts job concentration.” (Expert 8)
- “There are programs that require employees to completely alter their work methods, or they are underdeveloped…” (Expert 14)

Dependency on DICT was identified as a constituent of digital job demands in the workplace. The outcomes associated with digital job demands have been delineated in prior research and acknowledged by 12 experts.

#### 3.1.3. Work Intensification through DICT

An additional factor influencing job demands in relation to DICT usage is the escalation of work intensity. The utilization of DICT in the workplace can augment both the speed and volume of tasks, leading to what is known as work intensification [36,43,57,58,60].

According to Barber and Santuzzi [61], the increased adoption of technology in the workplace can result in intensified work for employees, giving rise to a phenomenon known as “tele-pressure”. This pressure arises when employees face additional work demands beyond their regular workload due to the utilization of DICT. Bulger et al. [53] highlighted that work intensification caused by DICT can diminish employees’ focus on their well-being and personal lives, potentially leading to workaholism. It is crucial to comprehend the reasons behind the intensification of work due to DICT.

The use of DICT in the workplace can contribute to work intensification through various means. Automation facilitated by DICT can enhance work processes in terms of speed and efficiency, but it can also lead to an increased workload and expectations for quicker turnaround times. DICT enables swift and efficient communication among employees; however, this can generate heightened expectations for immediate responses, creating a sense of urgency and stress. Additionally, the use of DICT may necessitate additional IT administration, maintenance, and support, further burdening employees’ workloads and increasing job demands. DICT can also be employed for digital surveillance, involving the tracking of employee activities and communication. This can instill a sense of pressure and raise job demands, as employees may feel constantly monitored and evaluated.

The accelerated output resulting from DICT contributes directly to the speeding up of workflow, thereby contributing to work intensification. However, other factors, such as heightened expectations for immediate responses and increased workloads due to IT administration, also contribute to this intensification.

- “Employees now have a much larger workload on the digital front.” (Expert 5)
- “There is a considerable increase in the amount of work, both in terms of quality and quantity. Digitization has tremendously expedited job processes.” (Expert 7)
- “The accelerating pace of work processes due to digital technologies, coupled with the growing expectation for faster work, leads to work intensification, which can have a negative impact on employee well-being and job satisfaction.” (Expert 9)
- “The job is becoming more condensed, which adds to the stress factor.” (Expert 13)

The experts highlighted the significant influence of DICT usage on work intensification, a phenomenon also documented in published literature. Work intensification resulting from DICT is recognized as a facet of digital job demands, as any innovative change in the realm of DICT affects working conditions.

#### 3.1.4. Other Aspects of Digital Job Demands

Additional aspects of digitization in the workplace were identified but not included in this study due to their lower combined scores. These aspects underscore the diverse impact of digitization in the workplace and its profound influence on job demands.

One aspect is the transformation of communication through DICT; for example, cyberaggression and ineffective communication can impact job demands [2,58]. Another challenge is information overload [62].

Another aspect described in the literature is job monitoring through DICT [36]. This technology can enhance management’s control over employees and influence job demands [58,62,63,64].

Digitization in the workplace can generate various forms of insecurity among employees. While insecurity can arise when one’s job is threatened by digitization, the introduction of new DICT can also lead employees to perceive technological uncertainty when dealing with these technologies [65].

Koen and Parker [66] stated that the possibility of implementing new technology can result in a chronically insecure work environment. These two factors influence job demands in different work contexts. Furthermore, the introduction of DICT is often linked to increased expectations for learning [58,62].

These aspects of digitization provide further insight into how digitization is reshaping and augmenting job demands in various ways. This brief overview highlights the transformative influence of digitization on job demands and working conditions.

### 3.2. Digital Job Resources

We identified aspects of digitization in the workplace for white-collar employees that serve as digital job resources. These aspects are beneficial in achieving job-related objectives and mitigating the psychological burdens of job demands through the utilization of DICT.

#### 3.2.1. Autonomy through DICT

An essential aspect of digitization in the workplace is the facilitation of autonomous or flexible work. Flexible job design refers to an approach that grants employees greater control over how they carry out their tasks. This approach entails a more autonomous structure of job performance, wherein employees have increased authority over their work schedules and task completion [43,62,63,64,67,68]. Other studies in this context have focused on enhanced flexibility regarding time and location [2,54,57].

Melzer and Diewald [64] highlighted that digitalized work systems enable timely and flexible decision making. These decisions can address the challenges posed by flexible and dynamic markets. Hoeven and Zoonen [43] explained that the implementation of DICT in the workplace allows for spatial and temporal flexibility.

All 15 experts described the use of DICT to promote location- and time-independent work, contributing to employees’ ability to develop autonomous working methods. Both of these aspects result in heightened job flexibility.

- “The advantage of this digital job is also that we can have much more flexibility in how we want to work.” (Expert 4)
- “I would contend that the digital environment allows more flexibility in general.” (Expert 11)
- “But you also have to remember that digitization has a lot of advantages because it naturally offers a lot of flexibility.” (Expert 14)
- “The relieving factor from my point of view is the flexibilization of job structures.” (Expert 15)

The utilization of DICT to promote autonomy is recognized as a digital job resource in the workplace. It facilitates flexible and autonomous work, thereby mitigating the psychological burdens associated with digital job demands.

#### 3.2.2. Collaboration through DICT

The opportunities provided by DICT for data and information sharing as a foundation for enhanced collaboration are invaluable, given that organizations make targeted use of them. Previous studies have discussed how the deliberate utilization of digital capabilities in the workplace can enhance collaboration [2,43,69].

Day et al. [2] asserted that DICT improves collaboration in the work environment by enhancing accessibility and availability. The portability of technological devices such as laptops and smartphones, coupled with real-time access to DICT functions such as emails and chat, enables employees to respond to inquiries even outside of traditional office hours. Wright et al. [69] emphasized that DICT facilitates collaboration beyond regular working hours.

This aspect of digitization was also recognized as a digital job resource in the expert interviews.

- “The ability to communicate asynchronously is a highly significant aspect.” (Expert 1)
- “Employees can communicate rapidly and with greater ease, even when remote.” (Expert 5)
- “In addition to facilitating swift communication, there is quick access to information.” (Expert 11)
- “Collaboration becomes feasible regardless of location, free from barriers, which is a genuine advantage.” (Expert 14)

DICT offers the potential to streamline and enhance collaboration compared to traditional methods. It facilitates real-time communication and information sharing regardless of employees’ physical locations, resulting in time savings and reduced reliance on face-to-face meetings. Digital collaboration also provides a more comprehensive and easily accessible record of shared information and communication. Ultimately, these advantages contribute to improved job performance and satisfaction. Twelve experts emphasized the impact of digitization as a resource in the workplace and highlighted the new opportunities it brings for collaboration.

#### 3.2.3. Efficiency through DICT

Day et al. [2] and Melzer and Diewald [64] recognized efficiency improvements as job resources. Day et al. [2] proposed that the effects of technology adoption serve as job resources. Melzer and Diewald [64] argued that the adoption of advanced technology can offer new resources that enhance job performance and satisfaction. Consequently, it is crucial for organizations to effectively manage the implementation of DICT.

A total of 14 experts highlighted the efficiency gained from DICT utilization as a resource in the workplace.

- “I believe that utilizing digital media in the correct manner results in improved efficiency.” (Expert 3)
- “Modern technology brings various benefits. Processes can become more efficient.” (Expert 13)
- “I strongly believe that we can also optimize workforce utilization through digitization.” (Expert 14)

The majority of experts and literature identified this aspect, prompting us to recognize efficiency through DICT as another component of digital job resources. When appropriately integrated into the work context, DICT can serve as a digital job resource that enhances efficiency.

#### 3.2.4. Other Aspects of Digital Job Resources

We have identified two additional digital job resources, but their combined scores were not relevant to this study. Variations in DICT and monitoring for support had low combined scores. However, they may hold greater relevance in other work contexts.

Variations in DICT encompass the different versions of computer programs, applications, or devices, such as computers, peripherals, and smart devices. The wide range of DICT variations provides employees with more options for task completion, which can enhance their job satisfaction and motivation. It grants greater control over work, enabling employees to choose when and where to complete tasks, leading to higher engagement and productivity [2].

Another classified digital job resource is monitoring support through DICT. DICT can be utilized to provide information and support to employers during the work process. Monitoring support refers to the use of technology to track and evaluate employee performance and provide assistance for improvement [2].

### 3.3. Digital Job Demands and Digital Job Resources in the JD-R Model

This triangulation study has identified the aspects of digitization that impact the work context of white-collar employees. Using the definitions of the JD-R model, we have categorized these aspects under the domains of job demands and job resources, thereby extending the JD-R model to encompass the effects of digitization in the workplace.

In the JD-R model, job demands refer to the physical, psychological, social, and organizational aspects of a job that typically require sustained physical or psychological effort [23]. We have expanded this category to include digital job demands, such as availability, dependence, and work intensification.

Job resources are defined as the working conditions that meet three criteria: being functionally necessary for achieving job-related goals, reducing the physical and psychological costs of job demands, and fostering personal growth and development [23]. This study has identified DICT-driven autonomy, collaboration, and efficiency as digital job resources.

**Figure 5:**
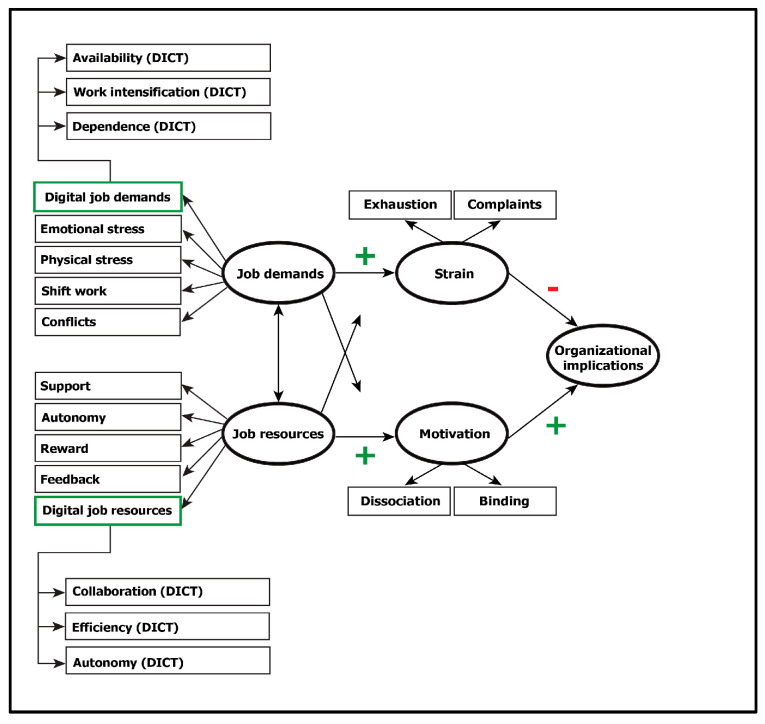
Extension of the JD-R model including digital job demands and digital job resources.

The integration of digital job demands and resources expands the JD-R model (Figure 5). Excessive job demands can lead to stress, which can have negative implications for employee motivation and well-being. Hence, stress should be considered as a potential factor when examining the impact of job demands and resources on employee outcomes. Job demands and resources can interact with each other over time, influencing employee motivation and well-being in complex ways. Thus, the multifaceted phenomenon of digitization can be comprehensively understood in terms of its organizational effects.

## 4. Discussion

This study aimed to examine the suitability of the JD-R model as a theoretical framework for understanding the complex effects of digitization in the workplace, using white-collar employees as a specific example. By dividing work conditions into job demands and resources within the JD-R model, we structured the contradictory effects of digitization and integrated these aspects as factors in both model constructs. This extension of the JD-R model provides a theoretical foundation for analyzing the dynamics of stress, motivation, and the organizational consequences of digitization. An extension of the JD-R model that includes digital job demands and digital job requirements provides a theoretical basis to broaden the discourse on technostress by bilaterally addressing the dark side and the bright side of digitization in organizations. Marsh et al. also highlighted the JD-R model as a useful theoretical foundation for exploring digitization in the work context. Understanding how technology can act both as a job demand that can lead to adverse health effects and as a job resource that can have motivational effects is more helpful than the concept of eustress. Eustress is not considered a helpful concept in common definitions of workplace stress [4]. In the context of technostress, techno-eustress is increasingly being discussed, by incorporating a so-called bright side of digitization effects [9]. In addition, the known negative effects of technostress factors are also being questioned. Zhao et al. show that the identified causes of technostress do not always or necessarily impact employees’ well-being negatively. The reason for this is that they are associated with different assessment results. The study shows that employees should be aware that technostress is not a bad thing if they deal with it positively [10]. Califf et al. also called for a rethinking of the negative and positive role of technostress in organizations [7]. In this context, Maier et al. emphasized the importance of trial-period technostress and emphasized the experience of stress during the adaptation of new technologies [6]. There is relative consensus among the most noteworthy technostress scientists that the knowledge about techno-eustress still needs to be expanded [8].

Digitization will continue to establish new advanced technologies [70], such as artificial intelligence, big data, or cloud computing, in the work context, which will have new effects on working conditions. Therefore, it is important to create a theoretical basis with the JD-R model to be able to assess future changes in working conditions in the context of technostress and health-oriented work design. Upcoming challenges exist, as shown by Anthony’s study, such as how knowledge work collides with analytic technologies and how aversion to algorithms drives black boxing owing to the information overload of a chronically uncertain work environment [71]. Bouncken et al.’s study represents another example; they exposed the connection of virtual collaboration and reduced absorptive learning capacity that leads to virtual traps and technostress [72].

The impact of digitization on organizations is significant. Mahapatra and Pillai showed that technostress is becoming a serious issue that negatively influences employee productivity, job satisfaction, and job engagement while increasing role stress, burnout, and exhaustion [73]. Marsh et al. presented six cognitive and affective outcomes (strain, work-family conflict, burnout, job satisfaction, end-user satisfaction, and well-being) and four behavioral outcomes (productivity, performance, organizational commitment, and turnover intentions) that are forced by digitization in the work context [4]. To summarize, digitization has created many challenges, as well as excellent opportunities, for organizations [11]. Digitization itself can act as a resource rather than a demand [4]. Organizations have the opportunity to turn digitalization and automation into a job resource, and thereby, mitigate the negative effects [12]. To take advantage of this, organizations must reach digital maturity, which means seizing digital opportunities, implementing effective strategies, and creating a humanized workplace [15].

The proposed extension of the JD-R model offers a starting point by providing a more comprehensive understanding of changes in job demands and resources in the context of digitization. This aligns with the original assertion by Demerouti and Nachreiner [33] that the model should be consistently developed and adapted to current work situations. It helps organizations to find a sustainable way to master digital transformation. To design digitization in the workplace for the future, it is crucial to analyze these diverse effects in a structured manner, as the effects of digitization vary depending on the specific work scenario. Moreover, every organization has a different digitization scenario; therefore, there is no one-size-fits-all approach to managing digitization [73].

The JD-R model provides a framework for systematically capturing the multifaceted characteristics and impacts of digitization in the workplace, while also allowing flexibility to apply the model across diverse job contexts and scenarios. The classification of digital job resources and digital job demands is a means to bring all the aspects of digitization together in a structured manner.

One limitation of this qualitative study is the potential for bias and subjectivity in data interpretation. Additionally, the sample size and representativeness of the experts may be a concern. This study focused on white-collar employees, limiting the generalizability of the findings to other work environments. Future studies should explore the impact of digitization on job demands and resources across different work scenarios and industries to gain a more comprehensive understanding of how digitization influences work in various settings.

Quantitative follow-up studies are necessary to empirically validate the conceptual extension of the JD-R model and quantify the holistic influence of digitization in the workplace. Previous studies have only examined partial aspects of digitization in the workplace, but the JD-R model offers the potential for a deeper understanding of digital workplace scenarios and systematically drawing conclusions for the design of future work environments. The results of this study provide a foundation for quantitative research to investigate the extent to which digital working conditions impact psychological strain and motivation in a work context. This study demonstrates the implementation of digital job demands and resources in the JD-R model and offers a theoretical framework to systematically investigate the complex effects of digitization in the workplace. This can facilitate targeted preventive health design for digitalized workplaces in various work scenarios. By analyzing the effects of motivation and psychological strain in digital work contexts, the variables digital job demands and digital job resources can be expanded to encompass new effects of digitization as new technologies emerge, providing guidance for understanding the effects in digital work scenarios.

In summary, organizations should embrace the possibilities of digitization to shape a positive future, taking proactive steps to prioritize digital health in the workplace. By understanding the impact of digitization in work scenarios and applying the JD-R model, organizations can utilize digital resources effectively, prevent job demands from becoming burdens, and ultimately make digitization a valuable job resource.

## 5. Conclusions

This study demonstrates the potential of using the JD-R model as a theoretical framework to incorporate job demands and job resources influenced by digitization into the future design of health-oriented jobs. The JD-R model provides a structured approach to considering the impact of digitization in the work context and deriving targeted preventive measures for employee health. While this study focused on white-collar employees, it is crucial to investigate other work scenarios to explore the effects of digitization on job demands and resources. The study highlights the diverse effects of digitization, emphasizing the need to systematize these effects and incorporate new effects arising from emerging digital technologies in future research.

By adopting a holistic perspective on the effects of digitization, organizations can develop effective strategies for managing the impact of digitization on job demands and resources, ultimately promoting employee well-being and performance.

Every organization should first understand how digitization affects working conditions in its own scenario and then plan which digital resources or digital job demands can be targeted to set up specific measures that will sustainably shape the organization and strengthen digital maturity. Therefore, it is essential that organizations see digitization as an opportunity in the face of increasing competition and, at the same time, consciously deal with the resulting risks. The goal of the organizations must be to orient the working world toward health in the long term, and thus, develop the working environment of employees in a sustainable manner. The aim is to reduce the negative effects of digitization, and thereby, fully utilize digitization as a resource.

Although the results of the current study are limited by its design, they provide a starting point for further systematizing the effect of digitization and for testing the extension of the JD-R model. Future studies should explore the comprehensive influence of digitization on job demands and resources across various work scenarios and industries to gain a thorough understanding of its effects. It is essential to validate the extension of the model through quantitative studies in different organizations to assess its applicability and validity.

## Data Availability

The data presented in this study are available on request from the corresponding author. The data are not publicly available due to privacy restrictions.
